# Identification of clinically actionable secondary genetic variants from whole‐genome sequencing in a large‐scale Chinese population

**DOI:** 10.1002/ctm2.866

**Published:** 2022-05-11

**Authors:** Pei‐Kuan Cong, Saber Khederzadeh, Cheng‐Da Yuan, Rui‐Jie Ma, Yi‐Yao Zhang, Jun‐Quan Liu, Shi‐Hui Yu, Lin Xu, Jian‐Hua Gao, Hong‐Xu Pan, Jin‐Chen Li, Shu‐Yang Xie, Ke‐Qi Liu, Bei‐Sha Tang, Hou‐Feng Zheng

**Affiliations:** ^1^ Diseases & Population (DaP) Geninfo Lab School of Life Sciences, Westlake University Hangzhou Zhejiang China; ^2^ Westlake Laboratory of Life Sciences and Biomedicine Hangzhou Zhejiang China; ^3^ Institute of Basic Medical Sciences, Westlake Institute for Advanced Study Hangzhou Zhejiang China; ^4^ Department of Dermatology Hangzhou Hospital of Traditional Chinese Medicine Hangzhou Zhejiang China; ^5^ Clinical Genome Center KingMed Diagnostics Co. Ltd. Guangzhou Guangdong China; ^6^ WBBC Shandong Center Binzhou Medical University Yantai Shandong China; ^7^ WBBC Jiangxi Center Jiangxi Medical College Shangrao Jiangxi China; ^8^ National Clinical Research Center for Geriatric Disorders Xiangya Hospital, Central South University Changsha Hunan China; ^9^ Department of Neurology Xiangya Hospital, Central South University Changsha Hunan China

To the Editor:

Clinical DNA sequencing is increasingly being chosen as a diagnostic test for Mendelian disorders in genomic medicine. Besides the primary findings, clinically actionable secondary genetic variants could be detected in the DNA sequencing. The genetic variants from genes proposed by American College of Medical Genetics and Genomics (ACMG) should be reported to clinician as secondary findings if the annotation suggested pathogenic or likely pathogenic.^1^ With the increasing application of DNA sequencing in the clinic, the ACMG updated the SF v3.0 list to 73 genes in 2021.^2^ Ethnic disparities exist in allele frequency of pathogenic variants. From the NHLBI Exome Sequencing Project (ESP), 0.7% and 0.5% of adults of European and African ancestry, respectively, were expected to have highly actionable penetrant pathogenic variants.^3^ Approximately 7% of 196 Korean individuals exhibited pathogenic variants,^4^ and at least one pathogenic variant was reported in 21% of 2049 Japanese individuals.^5^ The carrier frequency of secondary findings was highly variable among populations, but the prevalence of pathogenic or likely pathogenic variants (P/LP) in Chinese population remains unclear.

We analysed 4480 individuals’ whole‐genome sequencing data from Westlake BioBank for Chinese pilot project (WBBC)^6^, ^7^ to evaluate the prevalence of pathogenic genetic variants in the Chinese population for the 73 genes recommended by ACMG, and further investigated the ethnic differences among worldwide populations. A total of 9373 variants were found in the coding region, splicing site, intron and UTR in the WBBC samples, with 97.3% of these being missense and synonymous variants (Table S1). Following the variant classification standard (Figure 1 and Supporting Information), we identified 295 P/LP variants (99 pathogenic and 196 likely pathogenic variants, Table S2), accounting for 3.15% of the variants. For autosomal dominant inheritance (AD), the ratio of the P/LP variants was highest for *TNNT2* (24.14%), *LDLR* (21.65%) and *SCN5A* (14.69%) genes (Table S3). The highest ratio of the P/LP variants was shown by *MUTYH* (24.07%), *ATP7B* (23.93%) and *GAA* (12.93%) for the autosomal recessive inheritance (AR). Additionally, 20% (3/15) of the variants were P/LP variants in *GLA* (X‐linked inheritance) gene.

**FIGURE 1:**
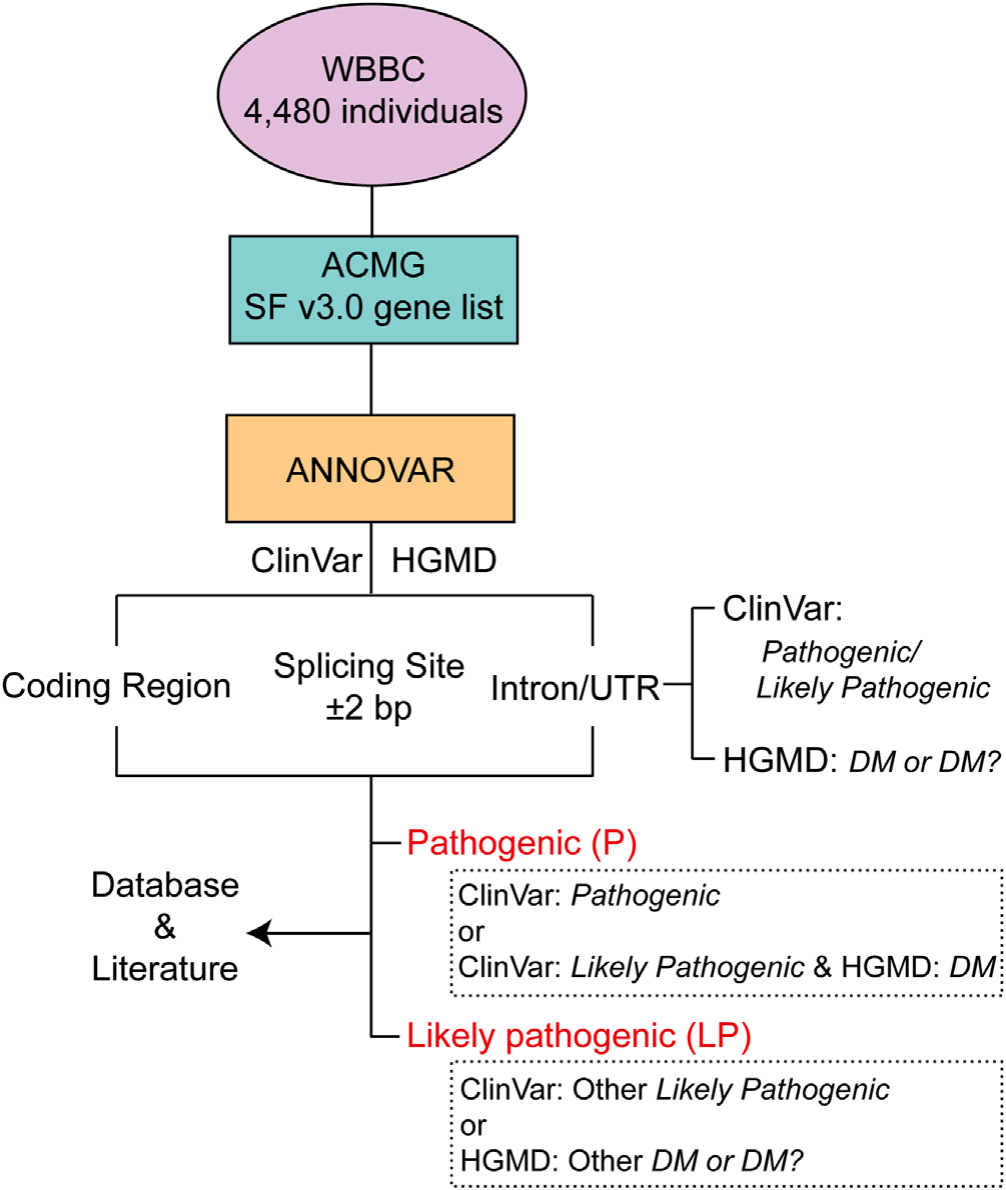
Scheme of pathogenic/likely pathogenic (P/LP) variants analysis pipeline. These variants were extracted from 4480 Chinese individuals in the WBBC project cohort. A total of 167 120 variants were annotated by the ANNOVAR, ClinVar and HGMD. The database HGMD Professional classified the pathogenic variants into disease‐causing or likely disease‐causing mutation (DM or DM?)

At the population level, approximately 17.37% (778/4480) of Chinese individuals carried at least one reported P/LP variant, whereas 4.2% (186/4480) of individuals had the pathogenic (P) variants. Because the 4480 samples also included individuals with Parkinson's disease (PD), we estimated a population frequency of 16.6% for P/LP variants in the PD patients and 18% in relatively healthy individuals. The proportion of P/LP carriers showed no significant differences between the PD patients and relatively healthy individuals (*p* = .297). Excluding the autosomal recessive condition carriers, the prevalence of P/LP variants was 10.9% (488/4480) compared to 1.4% (62/4480) for pathogenic variants in the WBBC cohort. For the autosomal dominant cardiovascular and cancer diseases, we found that 7.32% and 2.67% of the individuals carried P/LP variants in 31 cardiovascular and 27 cancer genes, respectively. A closer look at the single gene, *MUTYH* (3.15%, AR), *ATP7B* (2.86%, AR), *SCN5A* (1.96%, AD), *LDLR* (1.72%, AD) and *GAA* (1.03%, AR) showed a relatively high population frequency of the P/LP variants in the Chinese population (Table S3).

Our study observed significant ethnic differences in allele frequency of likely pathogenic or pathogenic variants between Chinese and European populations (Table 1 and Figure 2). We found that 24 P/LP variants from 15 genes exhibited relatively remarkable ethnic differences (Table 1). The minor allele frequencies of variants p.Pro5Gln (*MSH2*, Figure 2A), c.850‐2A>G (*MUTYH*, Figure 2B) and p.Ala1180Val (*SCN5A*, Figure 2D) in the WBBC were relatively higher than in non‐East Asian populations (Supporting Information). Contrastingly, p.Gly382Asp (*MUTYH*, Figure 2C), c.‐32‐13T>G (*GAA*, Figure 2E) and p.Asp444His (*BTD*, Figure 2F) showed a significantly high allele frequency in European population. We found an unusual difference in the pathogenic variant p.Asp444His in the *BTD* gene where the allele frequency exceeded 2% in South Asian, European and Admixed American populations (MAF__SAS_ = 0.035, MAF__EUR_ = 0.043 and MAF__AMR_ = 0.019). However, this variant was very rarely detected in the East Asian population (MAF__WBBC_ = 0.0006 and MAF__EAS_ = 0). In fact, the prevalence of biotinidase deficiency in East Asian (1/15 000 in Japanese and 1/620 400 in Chinese^8^) was lower than other ethnic groups (e.g., 1/9000 in Brazil^9^; please refer to the Supporting Information for more details). To access the full list of the variants, we provided a user‐friendly website to search for the annotation and frequency of variants in Chinese and other populations (https://wbbc.westlake.edu.cn/).

**TABLE 1:** List of pathogenic/likely pathogenic variants with remarkable ethnic differences in allele frequency between Chinese and European populations

**Gene**	**Transcript**	**cDNA**	**Protein**	**ID**	**WBBC**	**EAS**	**EUR**	**gnomAD**	**P/LP**	**Inheritance**	**Diseases**
*APC*	NM_000038.6	c.5912C>G	p.Ser1971Cys	rs754691867	0.0012	0	0	0.000065	LP	AD	Familial adenomatous polyposis
*APOB*	NM_000384.3	c.10579C>T	p.Arg3527Trp	rs144467873	0.0011	0.001	0	0.000065	LP	AD	Familial hypercholesterolemia
*ATP7B*	NM_000053.4	c.2333G>T	p.Arg778Leu	rs28942074	0.0018	0	0	0.000097	P	AR	Wilson's disease
*ATP7B*	NM_000053.4	c.2975C>T	p.Pro992Leu	rs201038679	0.0019	0	0	0.000032	P	AR	Wilson's disease
*ATP7B*	NM_000053.4	c.3316G>A	p.Val1106Ile	rs541208827	0.0018	0.002	0	0.0002	LP	AR	Wilson's disease
*ATP7B*	NM_000053.4	c.3443T>C	p.Ile1148Thr	rs60431989	0.0013	0	0	0.000032	P	AR	Wilson's disease
*BRCA2*	NM_000059.3	c.7088A>G	p.Tyr2363Cys	rs80358939	0.0009	0	0	0	LP	AD	Hereditary breast and ovarian cancer
*BTD*	NM_000060.4	c.1330G>C	p.Asp444His	rs13078881	0.0006	0	0.0427	0.0286	LP	AR	Biotinidase deficiency
*DSG2*	NM_001943.5	c.1592T>G	p.Phe531Cys	rs200484060	0.0016	0	0	0.000065	LP	AD	Arrhythmogenic right ventricular cardiomyopathy
*GAA*	NM_000152.5	c.2132C>G	p.Thr711Arg	rs759292700	0.0018	0	0	0.000032	LP	AR	Pompe disease
*GAA*	NM_000152.5	c.‐32‐13T>G	.	rs386834236	0.0003	0	0.007	0.003	P	AR	Pompe disease
*GLA*	NM_000169.3	c.1067G>A	p.Arg356Gln	rs869312163	0.0015	0	0	0	LP	XL	Fabry disease
*GLA*	NM_000169.3	c.640‐801G>A	.	rs199473684	0.0010	0	0	0.000046	P	XL	Fabry disease
*LDLR*	NM_000527.5	c.1765G>A	p.Asp589Asn	rs201971888	0.0015	0.003	0	0.000032	LP	AD	Familial hypercholesterolemia
*LDLR*	NM_000527.5	c.344G>A	p.Arg115His	rs201102461	0.0017	0.001	0	0.0001	LP	AD	Familial hypercholesterolemia
*LDLR*	NM_000527.5	c.769C>T	p.Arg257Trp	rs200990725	0.0015	0.003	0	0.000065	LP	AD	Familial hypercholesterolemia
*MSH2*	NM_000251.2	c.14C>A	p.Pro5Gln	rs56170584	0.0025	0.001	0	0	LP	AD	Lynch syndrome
*MSH2*	NM_000251.2	c.2516A>G	p.His839Arg	rs63750027	0.0012	0	0	0.000065	LP	AD	Lynch syndrome
*MUTYH*	NM_001048171.1	c.1145G>A	p.Gly382Asp	rs36053993	0.0002	0	0.0089	0.0032	P	AR	MUTYH‐associated polyposis
*MUTYH*	NM_001048171.1	c.850‐2A>G	.	rs77542170	0.0131	0.0149	0	0.0004	LP	AR	MUTYH‐associated polyposis
*MYBPC3*	NM_000256.3	c.2504G>T	p.Arg835Leu	rs527305885	0.0013	0.002	0	0.000065	LP	AD	Hypertrophic cardiomyopathy
*MYH7*	NM_000257.4	c.1322C>T	p.Thr441Met	rs121913653	0.0011	0	0	0.0002	LP	AD	Hypertrophic cardiomyopathy
*RYR1*	NM_000540.2	c.11518G>A	p.Val3840Ile	rs140616359	0.0010	0.001	0	0.000065	LP	AD	Malignant hyperthermia
*SCN5A*	NM_198056.2	c.3539C>T	p.Ala1180Val	rs41310765	0.0033	0.001	0	0.0002	LP	AD	Long QT syndrome 3

Abbreviations: EAS, the allele frequency of East Asian in the 1000 Genome Project; EUR, the allele frequency of European in the 1000 Genome Project; gnomAD, gnomAD_hg19_r211; Mode of inheritance, AD (autosomal dominant), AR (autosomal recessive) and XL (X‐linked); WBBC, the allele frequency of Chinese in the Westlake BioBank for Chinese.

**FIGURE 2:**
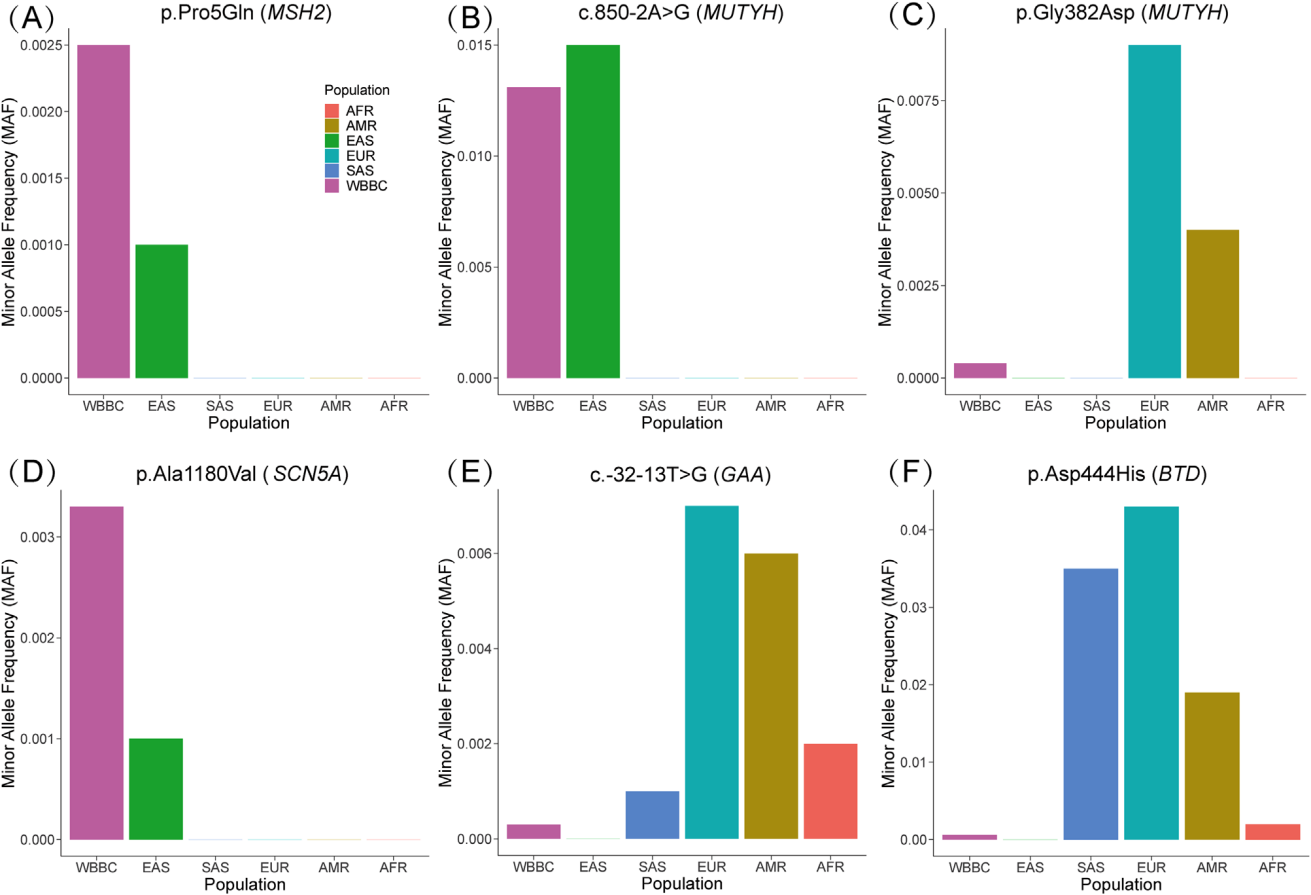
Comparison of the minor allele frequency of six variants in the WBBC and 1000 Genome Project. WBBC (Westlake BioBank for Chinese), EUR (European), EAS (East Asian), AMR (Admixed American), SAS (South Asian) and AFR (African)

Considering the ethnic discrepancies in incidence of diseases, the recommendation list should include highly penetrant phenotypes and genes in the East Asian population. Citrin deficiency, an inherited autosomal recessive metabolic disease, was initially reported and found mostly in individuals of East Asian ancestry.^10^ We found four heterozygous pathogenic variants of *SLC25A13*, c.550C>T (p.Arg184*), c.615+5G>A, c.852_855del and c.1180+1G>A in 1.5% (66/4480) in the individuals from WBBC. The c.852_855del variant in *SLC25A13* gene was the most common variants among East Asians (MAF__WBBC_ = 0.006 and MAF__EAS_ = 0.004) but rarely detected in other populations.

In conclusion, we found that approximately 17.37% (778/4480) of Chinese individuals carried at least one reported P/LP variant in the 73 genes recommended by ACMG, and 295 P/LP genetic variants were detected in our WBBC pilot cohort. We observed ethnic differences in allele frequency of P/LP variants between Chinese and European populations, 24 P/LP variants from 15 genes exhibited relatively remarkable ethnic differences (such as rs13078881 on *BTD* for biotinidase deficiency). We also suggested that high‐penetrance genes (e.g., *SLC25A13* gene for citrin deficiency) in the East Asians should be included in the recommendation list. Prevention and early intervention could reduce the risk of potentially severe consequences of genetic disorders for the undiagnosed carriers; therefore, secondary findings should be incorporated in clinical DNA sequencing reports appropriately.

## CONFLICT OF INTEREST

The authors declare that there is no conflict of interest.

## Supporting information

Supporting Information
