# Supplementary material for: Identification of clinically actionable secondary genetic variants from whole‐genome sequencing in a large‐scale Chinese population
Source: Clin Transl Med. 2022 May 11;12(5):e866. doi: 10.1002/ctm2.866 (PMC9091982; doi:10.1002/ctm2.866)
Supplement: Supplementary file 1 — Supporting Information [file CTM2-12-e866-s001.docx]

**Supporting Information**

**Supplementary Materials and Methods**

**Study participants and whole-genome sequencing**

Westlake BioBank for Chinese (WBBC) pilot cohort is a population-based prospective study with its major purpose to better understand the effect of genetic and environmental factors on growth and development from adolescents to adults.^1^ The WBBC pilot project was approved by the institutional review board of the Westlake University and Xiangya Hospital of Central South University. All members signed the consent forms. Initially, the WBBC conducted whole-genome sequencing in 4,535 individuals (13.9×) and whole-genome genotyping in 5,481 individuals from 29 out of 34 administrative divisions of China on NovaSeq 6000 platform.^2^ A total of 4,480 whole-genome sequencing individuals passed sample quality control for contamination and duplication, with 1,973 were Parkinson’s disease patients (mean age at 61.9), 1307 old participants (mean age at 62.4), and 1200 healthy adolescents (age ranging from 16 year to 25 year, 18.7 averagely). Individuals with severely genetic disorders were not included in the cohort, however, individuals with bone fracture and osteoporosis were not ruled out in the old participants. In total, 81,498,995 single-nucleotide variants (SNVs) and insertions and deletions (INDELs) were identified with the GATK4 variants calling pipeline.^2,3^ This variants set was used in downstream data analysis and statistics.

**Variant annotation**

We identified the variants in the 73 genes recommended by the ACMG SF v3.0 based on the genomic coordinates of the variants set on the chromosome. The ACMG SF v3.0 list reported 73 genes, including 28 genes related to 16 cancer phenotypes, 33 genes related to 10 cardiovascular phenotypes, 4 genes related to 4 inborn errors of metabolism phenotypes and 8 genes related to 6 miscellaneous phenotypes. A total of 167,120 variants, including 156,638 single-nucleotide variants (SNVs) and 10,482 insertions and deletions (INDELs), were identified in 4,480 Chinese individuals from the WBBC pilot cohort. The functional effects of variants were annotated using the ANNOVAR tool.^4^ The pathogenicity of missense variants was predicted by SIFT,^5^ Polyphen-2,^6^ MutationTaster,^7^ M-CAP^8^ and LRT.^9^ Allele frequency for each variant was obtained from WBBC^2^ and the 1000 Genome Project.^10^ Variant pathogenicity descriptions were searched from the ClinVar (release 20210814)^11^ and Human Gene Mutation Database (HGMD, version 2017.5).^12^ The clinical significance of the variants were categorized into “Pathogenic”, “Likely pathogenic”, “Conflicting interpretations of pathogenicity”, “Uncertain significance”, “Likely benign”, and “Benign” in the ClinVar database. The database HGMD^®^ Professional classified the variants into disease-causing mutation (DM), likely disease-causing mutation (DM?), disease-associated polymorphism (DP), in vitro or in vivo functional polymorphism (FP), and disease-associated polymorphism with additional functional evidence (DFP). We selected all variants in the coding region and splicing site (±2bp), and variants with the description of “Pathogenic/Likely Pathogenic” in the ClinVar or “DM/DM?” in the HGMD database as the candidate pathogenic variants in intronic and UTR regions.

**Variant classification**

Variants annotated as “Pathogenic” in the ClinVar, or “Likely pathogenic” in the ClinVar and “DM” in the HGMD were defined as the pathogenic (P) variants (Figure 1). Other “Likely pathogenic” variants in the ClinVar and other “DM/DM?” variants in the HGMD were classified as the potentially likely pathogenic (LP) variants. We manually inspected all the candidate variants for evidence of pathogenicity in the variant database (e.g. LOVD ^13,14^) and published literatures, according to standards and guidelines jointly recommended by the ACMG and the Association for Molecular Pathology (AMP).^15^ Variants identified as benign in some literature or laboratory report were excluded from the pathogenic or likely pathogenic variants.

**Supplementary Discussions**

Biotinidase deficiency (BD) is an autosomal recessive metabolic disease that prevents the body from recycling the vitamin biotin, causing profound and partial deficiency. The combined incidence of profound and partial deficiency is about 1 in 60,000 newborns, with the majority of patients being of European descent.^16^ The prevalence varies from 1/9,000 in Brazil,^17^ 1/47,486 in seven European countries^18^ to 1/150,000 in Japanese population.^19^ To analyze the results and follow up data of screening for newborn organic aciduria in Zhejiang province, 3 individuals had biotinidase deficiency/holocarboxylase synthetase deficiency (1:620,400) were found in 1,861,262 newborns. The prevalence of biotinidase deficiency in Zhejiang province was 1/620,400, which was far less than other ethnic groups.^20^ The variant p.Asp444His (rs13078881) was the leading cause of partial biotinidase deﬁciency (10 - 30% of mean normal serum biotinidase activity).^21^ Most reported individuals with partial biotinidase deficiency carried the variant p.Asp444His in one allele of the *BTD* gene in combination with another pathogenic variant in the other allele, causing severe deficiency of the biotinidase activity.^21^ Unexpectedly, a remarkable ethnic difference was observed in the allele frequency of variant p.Asp444His between Chinese and non-East Asian populations (Figure 2F and Table 3). The minor allele frequency of p.Asp444His was 0.0006 in Chinese population; however, it was more common variant in European (MAF__EUR_ = 0.0427), American (MAF__AMR_ = 0.019) and South Asian (MAF__SAS_ = 0.035) population.

The prevalence of Brugada syndrome was approximately 3 to 5 per 10,000 people. This condition occurs much more frequently in people of Asian ancestry, particularly in Japanese and Southeast Asian populations (18/10,000). Brugada syndrome in Asians was nine times more common than in Caucasians and 36 times more common than in Hispanics.^22^ Of the 211 variants found in *SCN5A* gene, 14.69% of variants were considered to be the P/LP variants. The likely pathogenic missense variant p.Ala1180Val (rs41310765) in the *SCN5A* gene was identified in 30 heterozygous individuals (Figure 2D, MAF__WBBC_ = 0.0033). This variant was previously reported in patients with dilated cardiomyopathy ^23^ and co-segregated with the long QT syndrome in another Chinese family.^24^

The incidence of Pompe disease was estimated at one in 40,000 people in the Netherlands^25^ and New York.^26^ For the infantile-onset Pompe disease, East Asian had higher proportions of pathogenic or likely pathogenic variants.^27^ The predicted genetic prevalence was observed in the East Asian population at 1:12,125, Non-Finnish European (1:13,756), Ashkenazi Jewish (1:22,851), African/African-American (1:26,560), Latino/Admixed American (1:57,620), South Asian (1:93,087), and Finnish (1:1,056,444).^27^ The c.-32-13T>G (*GAA*) was the most frequent mutation and accounted for 36 - 90% of late-onset Pompe disease,^28,29^ which resulted in exon 2 skipping during pre-mRNA splicing.^30^ However, the allele frequency of this variant in East Asian was lower than European population.

In our study, we found 39 P/LP variants in the *ATP7B* gene associated with Wilson’s disease (WD). A significant proportion (2.86%) of individuals carried at least one allele of P/LP variants in the *ATP7B* gene. The allele frequency of missense variants p.Arg778Leu (MAF = 0.0018), p.Pro992Leu (MAF = 0.0019), p.Val1106Ile (MAF = 0.0018) and p.Ile1148Thr (MAF = 0.0013) in the Chinese population were relatively higher than in the European population (Table 1). The variant p.Arg778Leu and p.Pro992Leu were also the most frequent pathogenic variants in the *ATP7B* gene in the Chinese Wilson’s disease, accounting for 31.80% and 18.54%, respectively.^31^ In fact, the prevalence of WD is higher in China than in western countries.^32^ The prevalence of WD is estimated to be 1/100,000 to 1/30,000 worldwide.^33,34^ Hu et al reported that the prevalence of WD was 6.21/100,000 in Anhui Province.^35^

Sudden cardiac death is an unexpected death caused by loss of heart function and is estimated to account for over 544,000 deaths in China annually.^36^ The worldwide incidence of autosomal dominant cardiovascular phenotypes (Table S3) was 1.04%, including Aortopathies (1/5,000), ^37^ Arrhythmogenic right ventricular cardiomyopathy (1/5,000),^38^ Catecholaminergic polymorphic ventricular tachycardia (1/10,000),^39^ Dilated cardiomyopathy (1/250),^38^ Ehlers-Danlos syndrome (1/5,000),^40^ Familial hypercholesterolemia (1/300),^41^ Hypertrophic cardiomyopathy (1/500)^42^ and Long QT syndrome (1/2,500),^43^ respectively. We found that 7.32% of the Chinese individuals carried the P/LP variants. The prevalence of cardiovascular disorders was underestimated in Chinese population. Many cases were undiagnosed and phenotype healthy individuals might be potential patients. Prevention and early intervention can reduce the risk of potentially severe consequences for the undiagnosed patients of cardiovascular disorders.

**References**

1. Zhu XW, Liu KQ, Wang PY, et al. Cohort profile: the Westlake BioBank for Chinese (WBBC) pilot project. *BMJ Open.* 2021;11:e045564.

2. Peikuan C, Weiyang B, Jinchen L, et al. Genomic analyses of 10,376 individuals in the Westlake BioBank for Chinese (WBBC) pilot project. *Nature Portfolio.* 2021.

3. Van der Auwera GA, Carneiro MO, Hartl C, et al. From FastQ data to high confidence variant calls: the Genome Analysis Toolkit best practices pipeline. *Curr Protoc Bioinformatics.* 2013;43:11 10 11-11 10 33.

4. Wang K, Li M, Hakonarson H. ANNOVAR: functional annotation of genetic variants from high-throughput sequencing data. *Nucleic Acids Res.* 2010;38:e164.

5. Kumar P, Henikoff S, Ng PC. Predicting the effects of coding non-synonymous variants on protein function using the SIFT algorithm. *Nat Protoc.* 2009;4:1073-1081.

6. Adzhubei I, Jordan DM, Sunyaev SR. Predicting functional effect of human missense mutations using PolyPhen-2. *Curr Protoc Hum Genet.* 2013;Chapter 7:Unit7 20.

7. Schwarz JM, Cooper DN, Schuelke M, et al. MutationTaster2: mutation prediction for the deep-sequencing age. *Nat Methods.* 2014;11:361-362.

8. Jagadeesh KA, Wenger AM, Berger MJ, et al. M-CAP eliminates a majority of variants of uncertain significance in clinical exomes at high sensitivity. *Nat Genet.* 2016;48:1581-1586.

9. Chun S, Fay JC. Identification of deleterious mutations within three human genomes. *Genome Res.* 2009;19:1553-1561.

10. Genomes Project C, Auton A, Brooks LD, et al. A global reference for human genetic variation. *Nature.* 2015;526:68-74.

11. Landrum MJ, Lee JM, Benson M, et al. ClinVar: public archive of interpretations of clinically relevant variants. *Nucleic Acids Res.* 2016;44:D862-868.

12. Stenson PD, Mort M, Ball EV, et al. The Human Gene Mutation Database: 2008 update. *Genome Med.* 2009;1:13.

13. Zhang T, Moss A, Cong P, et al. LQTS gene LOVD database. *Hum Mutat.* 2010;31:E1801-1810.

14. Pan M, Cong P, Wang Y, et al. Novel LOVD databases for hereditary breast cancer and colorectal cancer genes in the Chinese population. *Hum Mutat.* 2011;32:1335-1340.

15. Richards S, Aziz N, Bale S, et al. Standards and guidelines for the interpretation of sequence variants: a joint consensus recommendation of the American College of Medical Genetics and Genomics and the Association for Molecular Pathology. *Genet Med.* 2015;17:405-424.

16. Wolf B. Worldwide survey of neonatal screening for biotinidase deficiency. *J Inherit Metab Dis.* 1991;14:923-927.

17. Neto EC, Schulte J, Rubim R, et al. Newborn screening for biotinidase deficiency in Brazil: biochemical and molecular characterizations. *Braz J Med Biol Res.* 2004;37:295-299.

18. Loeber JG. Neonatal screening in Europe; the situation in 2004. *J Inherit Metab Dis.* 2007;30:430-438.

19. Yamaguchi S. Newborn screening in Japan: restructuring for the new era. *Ann Acad Med Singap.* 2008;37:13-15.

20. Hong F, Huang X, Zhang Y, et al. [Screening for newborn organic aciduria in Zhejiang province:prevalence, outcome and follow-up]. *Zhejiang Da Xue Xue Bao Yi Xue Ban.* 2017;46:240-247.

21. Swango KL, Demirkol M, Huner G, et al. Partial biotinidase deficiency is usually due to the D444H mutation in the biotinidase gene. *Hum Genet.* 1998;102:571-575.

22. Vutthikraivit W, Rattanawong P, Putthapiban P, et al. Worldwide Prevalence of Brugada Syndrome: A Systematic Review and Meta-Analysis. *Acta Cardiol Sin.* 2018;34:267-277.

23. Ge J, Sun A, Paajanen V, et al. Molecular and clinical characterization of a novel SCN5A mutation associated with atrioventricular block and dilated cardiomyopathy. *Circ Arrhythm Electrophysiol.* 2008;1:83-92.

24. Zhang Y, Wang J, Chang S, et al. The SCN5A mutation A1180V is associated with electrocardiographic features of LQT3. *Pediatr Cardiol.* 2014;35:295-300.

25. Ausems MG, Verbiest J, Hermans MP, et al. Frequency of glycogen storage disease type II in The Netherlands: implications for diagnosis and genetic counselling. *Eur J Hum Genet.* 1999;7:713-716.

26. Martiniuk F, Chen A, Mack A, et al. Carrier frequency for glycogen storage disease type II in New York and estimates of affected individuals born with the disease. *Am J Med Genet.* 1998;79:69-72.

27. Park KS. Carrier frequency and predicted genetic prevalence of Pompe disease based on a general population database. *Mol Genet Metab Rep.* 2021;27:100734.

28. Hermans MM, van Leenen D, Kroos MA, et al. Twenty-two novel mutations in the lysosomal alpha-glucosidase gene (GAA) underscore the genotype-phenotype correlation in glycogen storage disease type II. *Hum Mutat.* 2004;23:47-56.

29. Montalvo AL, Bembi B, Donnarumma M, et al. Mutation profile of the GAA gene in 40 Italian patients with late onset glycogen storage disease type II. *Hum Mutat.* 2006;27:999-1006.

30. Bergsma AJ, Kroos M, Hoogeveen-Westerveld M, et al. Identification and characterization of aberrant GAA pre-mRNA splicing in pompe disease using a generic approach. *Hum Mutat.* 2015;36:57-68.

31. Cheng N, Wang H, Wu W, et al. Spectrum of ATP7B mutations and genotype-phenotype correlation in large-scale Chinese patients with Wilson Disease. *Clin Genet.* 2017;92:69-79.

32. Xie JJ, Wu ZY. Wilson's Disease in China. *Neurosci Bull.* 2017;33:323-330.

33. Reilly M, Daly L, Hutchinson M. An epidemiological study of Wilson's disease in the Republic of Ireland. *J Neurol Neurosurg Psychiatry.* 1993;56:298-300.

34. Coffey AJ, Durkie M, Hague S, et al. A genetic study of Wilson's disease in the United Kingdom. *Brain.* 2013;136:1476-1487.

35. Hu W, Han Y, Suo B, et al. Epidemiological study of hepatolenticular degeneration at Hanshan County, Anhui Province. *National Medical Journal of China.* 2011;91.

36. Zhang S. Sudden cardiac death in China. *Pacing Clin Electrophysiol.* 2009;32:1159-1162.

37. Paterick TE, Humphries JA, Ammar KA, et al. Aortopathies: etiologies, genetics, differential diagnosis, prognosis and management. *Am J Med.* 2013;126:670-678.

38. McKenna WJ, Judge DP. Epidemiology of the inherited cardiomyopathies. *Nat Rev Cardiol.* 2021;18:22-36.

39. Lieve KV, van der Werf C, Wilde AA. Catecholaminergic Polymorphic Ventricular Tachycardia. *Circ J.* 2016;80:1285-1291.

40. Alomari M, Hitawala A, Chadalavada P, et al. Prevalence and Predictors of Gastrointestinal Dysmotility in Patients with Hypermobile Ehlers-Danlos Syndrome: A Tertiary Care Center Experience. *Cureus.* 2020;12:e7881.

41. Beheshti SO, Madsen CM, Varbo A, et al. Worldwide Prevalence of Familial Hypercholesterolemia: Meta-Analyses of 11 Million Subjects. *J Am Coll Cardiol.* 2020;75:2553-2566.

42. Authors/Task Force m, Elliott PM, Anastasakis A, et al. 2014 ESC Guidelines on diagnosis and management of hypertrophic cardiomyopathy: the Task Force for the Diagnosis and Management of Hypertrophic Cardiomyopathy of the European Society of Cardiology (ESC). *Eur Heart J.* 2014;35:2733-2779.

43. Schwartz PJ, Stramba-Badiale M, Crotti L, et al. Prevalence of the congenital long-QT syndrome. *Circulation.* 2009;120:1761-1767.

**Table S1**. Summary of the variants in the 73 genes recommended by the ACMG SF v3.0 list in 4480 Chinese individuals

| **Function** | **Variants** | **P Variants** | **P/LP Variant** | **Ratio of P/LP** |
| --- | --- | --- | --- | --- |
| missense | 5,810 | 56 | 244 | 0.0420 |
| synonymous | 3,309 | 1 | 2 | 0.0006 |
| splicing | 42 | 7 | 11 | 0.2619 |
| frameshift deletion | 36 | 5 | 5 | 0.1389 |
| frameshift insertion | 21 | 4 | 4 | 0.1905 |
| nonframeshift deletion | 52 | 0 | 1 | 0.0192 |
| nonframeshift insertion | 18 | 0 | 1 | 0.0556 |
| stopgain | 75 | 26 | 27 | 0.3600 |
| stoploss | 2 | 0 | 0 | 0.0000 |
| startloss | 8 | 0 | 0 | 0.0000 |
| **Total** | **9,373** | **99** | **295** | **0.0315** |

T**able S2.** Reported pathogenic/likely pathogenic variants in the recommendation genes in the WBBC cohort

| Position | Ref | Alt | ID | Gene | Transcript | cDNA | Protein | WBBC | P/LP |
| --- | --- | --- | --- | --- | --- | --- | --- | --- | --- |
| 10_90697868 | G | A | rs886038978 | ACTA2 | NM_001613.4 | c.940C>T | p.Arg314* | 0.0001 | P |
| 10_90701048 | C | T | rs1057521105 | ACTA2 | NM_001613.4 | c.554G>A | p.Arg185Gln | 0.0001 | LP |
| 12_52309266 | T | C | rs1592224412 | ACVRL1 | NM_000020.3 | c.1030T>C | p.Cys344Arg | 0.0001 | P |
| 5_112175346 | T | C | rs528724202 | APC | NM_000038.6 | c.4055T>C | p.Val1352Ala | 0.0009 | LP |
| 5_112177203 | C | G | rs754691867 | APC | NM_000038.6 | c.5912C>G | p.Ser1971Cys | 0.0012 | LP |
| 2_21225140 | C | T | rs533755016 | APOB | NM_000384.3 | c.13154G>A | p.Arg4385His | 0.0001 | LP |
| 2_21228437 | A | G | rs376825639 | APOB | NM_000384.3 | c.11303T>C | p.Ile3768Thr | 0.0002 | LP |
| 2_21229040 | G | A | rs368278927 | APOB | NM_000384.3 | c.10700C>T | p.Thr3567Met | 0.0001 | LP |
| 2_21229161 | G | A | rs144467873 | APOB | NM_000384.3 | c.10579C>T | p.Arg3527Trp | 0.0011 | LP |
| 2_21232203 | G | A | rs146538280 | APOB | NM_000384.3 | c.7537C>T | p.Arg2513* | 0.0001 | P |
| 2_21252772 | G | A | rs771541567 | APOB | NM_000384.3 | c.1468C>T | p.Arg490Trp | 0.0001 | LP |
| 13_52509739 | G | A | rs755584106 | ATP7B | NM_000053.4 | c.4114C>T | p.Gln1372* | 0.0003 | P |
| 13_52511473 | C | G | rs778732681 | ATP7B | NM_000053.4 | c.3960G>C | p.Arg1320Ser | 0.0003 | P |
| 13_52511629 | C | T | rs199821556 | ATP7B | NM_000053.4 | c.3886G>A | p.Asp1296Asn | 0.0001 | LP |
| 13_52511631 | G | A | rs1340942427 | ATP7B | NM_000053.4 | c.3884C>T | p.Ala1295Val | 0.0001 | P |
| 13_52511656 | C | T | rs762866453 | ATP7B | NM_000053.4 | c.3859G>A | p.Gly1287Ser | 0.0004 | LP |
| 13_52511706 | T | C | rs121907990 | ATP7B | NM_000053.4 | c.3809A>G | p.Asn1270Ser | 0.0001 | P |
| 13_52511799 | A | C | rs374628199 | ATP7B | NM_000053.4 | c.3716T>G | p.Val1239Gly | 0.0001 | LP |
| 13_52513198 | T | C | rs200911496 | ATP7B | NM_000053.4 | c.3688A>G | p.Ile1230Val | 0.0001 | LP |
| 13_52515241 | T | C | . | ATP7B | NM_000053.4 | c.3532A>G | p.Thr1178Ala | 0.0001 | P |
| 13_52515306 | C | T | rs773917820 | ATP7B | NM_000053.4 | c.3467G>A | p.Arg1156His | 0.0001 | LP |
| 13_52515322 | G | A | rs755554442 | ATP7B | NM_000053.4 | c.3451C>T | p.Arg1151Cys | 0.0001 | P |
| 13_52515330 | A | G | rs60431989 | ATP7B | NM_000053.4 | c.3443T>C | p.Ile1148Thr | 0.0013 | P |
| 13_52515347 | C | G | rs778749563 | ATP7B | NM_000053.4 | c.3426G>C | p.Gln1142His | 0.0001 | LP |
| 13_52516618 | C | T | rs541208827 | ATP7B | NM_000053.4 | c.3316G>A | p.Val1106Ile | 0.0018 | LP |
| 13_52518318 | A | G | rs373601229 | ATP7B | NM_000053.4 | c.3170T>C | p.Leu1057Prp | 0.0001 | LP |
| 13_52518348 | T | A | . | ATP7B | NM_000053.4 | c.3140A>T | p.Asp1047Val | 0.0002 | P |
| 13_52520427 | G | A | rs371840514 | ATP7B | NM_000053.4 | c.3053C>T | p.Ala1018Val | 0.0002 | P |
| 13_52520439 | G | A | . | ATP7B | NM_000053.4 | c.3041C>T | p.Pro1014Leu | 0.0002 | LP |
| 13_52520472 | G | A | rs775055397 | ATP7B | NM_000053.4 | c.3008C>T | p.Ala1003Val | 0.0001 | P |
| 13_52520473 | C | T | rs201497300 | ATP7B | NM_000053.4 | c.3007G>A | p.Ala1003Thr | 0.0002 | P |
| 13_52520505 | G | A | rs201038679 | ATP7B | NM_000053.4 | c.2975C>T | p.Pro992Leu | 0.0019 | P |
| 13_52520541 | C | T | rs1038582488 | ATP7B | NM_000053.4 | c.2939G>A | p.Cys980Tyr | 0.0001 | P |
| 13_52520556 | G | T | rs778163447 | ATP7B | NM_000053.4 | c.2924C>A | p.Ser975Tyr | 0.0001 | P |
| 13_52520559 | G | A | rs201061621 | ATP7B | NM_000053.4 | c.2921C>T | p.Thr974Met | 0.0002 | LP |
| 13_52520575 | G | A | rs774028495 | ATP7B | NM_000053.4 | c.2905C>T | p.Arg969Trp | 0.0001 | P |
| 13_52523836 | C | T | rs28942076 | ATP7B | NM_000053.4 | c.2827G>A | p.Gly943Ser | 0.0001 | P |
| 13_52523908 | G | C | rs121907993 | ATP7B | NM_000053.4 | c.2755C>G | p.Arg919Gly | 0.0007 | P |
| 13_52524211 | T | G | rs1455758826 | ATP7B | NM_000053.4 | c.2662A>C | p.Thr888Pro | 0.0001 | P |
| 13_52524268 | C | T | rs191312027 | ATP7B | NM_000053.4 | c.2605G>A | p.Gly869Arg | 0.0015 | LP |
| 13_52532469 | C | A | rs28942074 | ATP7B | NM_000053.4 | c.2333G>T | p.Arg778Leu | 0.0018 | P |
| 13_52532470 | G | A | rs137853284 | ATP7B | NM_000053.4 | c.2332C>T | p.Arg778Trp | 0.0001 | P |
| 13_52532508 | T | C | rs1555291147 | ATP7B | NM_000053.4 | c.2294A>G | p.Asp765Gly | 0.0004 | P |
| 13_52532617 | T | C | rs773447981 | ATP7B | NM_000053.4 | c.2185A>G | p.Met729Val | 0.0002 | LP |
| 13_52532670 | C | T | . | ATP7B | NM_000053.4 | c.2132G>A | p.Gly711Glu | 0.0001 | LP |
| 13_52539174 | A | C | rs770829226 | ATP7B | NM_000053.4 | c.1708-5T>G | . | 0.0001 | P |
| 13_52544640 | G | A | rs1449610384 | ATP7B | NM_000053.4 | c.1531C>T | p.Gln511* | 0.0001 | P |
| 13_52548768 | G | T | rs756718353 | ATP7B | NM_000053.4 | c.588C>A | p.Asp196Glu | 0.0003 | LP |
| 13_52548830 | C | CT | rs758115611 | ATP7B | NM_000053.4 | c.525dupA | p.Val176Serfs*28 | 0.0001 | P |
| 13_52549234 | T | C | rs201738967 | ATP7B | NM_000053.4 | c.122A>G | p.Asn41Ser | 0.0002 | P |
| 17_41215957 | C | G | rs80357125 | BRCA1 | NM_007294.4 | c.5086G>C | p.Val1696Leu | 0.0001 | LP |
| 17_41223218 | GA | G | rs886037790 | BRCA1 | NM_007294.4 | c.4712delT | p.Phe1571Serfs*30 | 0.0001 | P |
| 17_41245067 | T | G | rs397508970 | BRCA1 | NM_007294.4 | c.2481A>C | p.Glu827Asp | 0.0003 | LP |
| 13_32899249 | G | A | rs80358603 | BRCA2 | NM_000059.3 | c.353G>A | p.Arg118His | 0.0001 | LP |
| 13_32906495 | G | T | rs397508009 | BRCA2 | NM_000059.3 | c.880G>T | p.Glu294* | 0.0001 | P |
| 13_32906565 | C | CA | rs80359770 | BRCA2 | NM_000059.3 | c.956dupA | p.Asn319Lysfs*8 | 0.0001 | P |
| 13_32907378 | A | G | rs373400041 | BRCA2 | NM_000059.3 | c.1763A>G | p.Asn588Ser | 0.0001 | LP |
| 13_32907420 | GA | G | rs80359307 | BRCA2 | NM_000059.3 | c.1813delA | p.Ile605Tyrfs*9 | 0.0002 | P |
| 13_32911601 | C | T | rs80358557 | BRCA2 | NM_000059.3 | c.3109C>T | p.Gln1037* | 0.0001 | P |
| 13_32913729 | C | CT | rs80359499 | BRCA2 | NM_000059.3 | c.5238dupT | p.Asn1747* | 0.0001 | P |
| 13_32913836 | C | T | rs80358757 | BRCA2 | NM_000059.3 | c.5344C>T | p.Gln1782* | 0.0001 | P |
| 13_32914461 | A | C | rs148618542 | BRCA2 | NM_000059.3 | c.5969A>C | p.Asp1990Ala | 0.0002 | LP |
| 13_32920978 | C | T | rs80358920 | BRCA2 | NM_000059.3 | c.6952C>T | p.Arg2318* | 0.0001 | P |
| 13_32929078 | A | G | rs80358939 | BRCA2 | NM_000059.3 | c.7088A>G | p.Tyr2363Cys | 0.0009 | LP |
| 13_32944563 | G | A | rs80359077 | BRCA2 | NM_000059.3 | c.8356G>A | p.Ala2786Thr | 0.0002 | LP |
| 13_32945123 | A | G | rs80359103 | BRCA2 | NM_000059.3 | c.8518A>G | p.Ile2840Val | 0.0001 | LP |
| 13_32972548 | C | T | rs770868371 | BRCA2 | NM_000059.3 | c.9898C>T | p.Pro3300Ser | 0.0001 | LP |
| 3_15643400 | A | T | rs143058480 | BTD | NM_000060.4 | c.43A>T | p.Arg15* | 0.0001 | LP |
| 3_15685958 | G | A | rs397514375 | BTD | NM_000060.4 | c.595G>A | p.Val199Met | 0.0001 | P |
| 3_15686634 | G | C | rs397514335 | BTD | NM_000060.4 | c.1271G>C | p.Cys424Ser | 0.0001 | P |
| 3_15686693 | G | C | rs13078881 | BTD | NM_000060.4 | c.1330G>C | p.Asp444His | 0.0006 | LP |
| 3_15686853 | C | CT | rs397514425 | BTD | NM_000060.4 | c.1493dupT | p.Leu498Phefs*13 | 0.0003 | P |
| 1_116283388 | G | A | rs775663612 | CASQ2 | NM_001232.3 | c.381C>T | p.Gly127Gly | 0.0001 | LP |
| 18_29099821 | G | A | rs121913008 | DSG2 | NM_001943.5 | c.137G>A | p.Arg46Gln | 0.0001 | P |
| 18_29116333 | T | G | rs200484060 | DSG2 | NM_001943.5 | c.1592T>G | p.Phe531Cys | 0.0016 | LP |
| 6_7580621 | C | T | rs770873593 | DSP | NM_004415.4 | c.4198C>T | p.Arg1400* | 0.0001 | P |
| 9_130579458 | G | A | rs764262721 | ENG | NM_000118.3 | c.1711C>T | p.Arg571Cys | 0.0001 | LP |
| 15_48703309 | T | C | rs376933421 | FBN1 | NM_000138.4 | c.8494A>G | p.Ser2832Gly | 0.0001 | LP |
| 15_48704869 | T | C | rs759494825 | FBN1 | NM_000138.4 | c.8123A>G | p.Asn2708Ser | 0.0001 | LP |
| 15_48714160 | G | A | rs763759308 | FBN1 | NM_000138.4 | c.7559C>T | p.Thr2520Met | 0.0006 | LP |
| 15_48718025 | C | T | rs143863014 | FBN1 | NM_000138.4 | c.7241G>A | p.Arg2414Gln | 0.0003 | LP |
| 15_48779517 | G | A | rs539103389 | FBN1 | NM_000138.4 | c.3455C>T | p.Ala1152Val | 0.0001 | LP |
| 15_48782087 | C | T | rs55831697 | FBN1 | NM_000138.4 | c.3043G>A | p.Ala1015Thr | 0.0001 | LP |
| 15_48787384 | T | G | rs770290542 | FBN1 | NM_000138.4 | c.2613A>C | p.Leu871Phe | 0.0002 | P |
| 15_48796041 | C | T | rs377621293 | FBN1 | NM_000138.4 | c.2056G>A | p.Ala686Thr | 0.0001 | LP |
| 15_48829825 | C | T | rs768744583 | FBN1 | NM_000138.4 | c.719G>A | p.Arg240His | 0.0001 | LP |
| 17_78078341 | T | G | rs386834236 | GAA | NM_000152.5 | c.-32-13T>G |  | 0.0003 | P |
| 17_78078626 | C | T | rs1555598687 | GAA | NM_000152.5 | c.241C>T | p.Gln81* | 0.0001 | P |
| 17_78078888 | G | A | rs376685205 | GAA | NM_000152.5 | c.503G>A | p.Arg168Gln | 0.0003 | LP |
| 17_78078918 | G | A | rs762267535 | GAA | NM_000152.5 | c.533G>A | p.Arg178His | 0.0001 | LP |
| 17_78081459 | C | T | rs1555599667 | GAA | NM_000152.5 | c.796C>T | p.Pro266Ser | 0.0001 | P |
| 17_78082312 | G | A | . | GAA | NM_000152.5 | c.1100G>A | p.Trp367* | 0.0001 | P |
| 17_78083849 | G | A | rs778068209 | GAA | NM_000152.5 | c.1432G>A | p.Gly478Arg | 0.0001 | P |
| 17_78085814 | A | T | rs747150965 | GAA | NM_000152.5 | c.1669A>T | p.Ile557Phe | 0.0001 | LP |
| 17_78086465 | G | A | rs549029029 | GAA | NM_000152.5 | c.1843G>A | p.Gly615Arg | 0.0001 | P |
| 17_78086744 | C | A | rs763456921 | GAA | NM_000152.5 | c.1958C>A | p.Thr653Asn | 0.0001 | LP |
| 17_78086806 | CACA | C | rs786204621 | GAA | NM_000152.5 | c.2024_2026delACA | p.Asn675del | 0.0001 | LP |
| 17_78087080 | C | T | rs786204645 | GAA | NM_000152.5 | c.2104C>T | p.Arg702Cys | 0.0001 | P |
| 17_78087108 | C | G | rs759292700 | GAA | NM_000152.5 | c.2132C>G | p.Thr711Arg | 0.0018 | LP |
| 17_78090813 | T | G | rs1479740763 | GAA | NM_000152.5 | c.2236T>G | p.Trp746Gly | 0.0006 | LP |
| 17_78090814 | G | C | rs752921215 | GAA | NM_000152.5 | c.2237G>C | p.Trp746Ser | 0.0002 | P |
| 17_78090815 | G | C | rs1800312 | GAA | NM_000152.5 | c.2238G>C | p.Trp746Cys | 0.0004 | P |
| 17_78092118 | C | T | rs780321415 | GAA | NM_000152.5 | c.2608C>T | p.Arg870* | 0.0001 | P |
| 17_78092467 | G | T | rs765718882 | GAA | NM_000152.5 | c.2662G>T | p.Glu888* | 0.0001 | P |
| 17_78093082 | CTG | C | rs1057517308 | GAA | NM_000152.5 | c.2815_2816delTG | p.Val939Leufs*78 | 0.0001 | P |
| X_100652915 | T | G | . | GLA | NM_000169.3 | c.1172A>C | p.Lys391Thr | 0.0005 | LP |
| X_100653020 | C | T | rs869312163 | GLA | NM_000169.3 | c.1067G>A | p.Arg356Gln | 0.0015 | LP |
| X_100654735 | C | T | rs199473684 | GLA | NM_000169.3 | c.640-801G>A |  | 0.0010 | P |
| 12_121416600 | C | T | rs774637975 | HNF1A | NM_000545.6 | c.29C>T | p.Thr10Met | 0.0003 | LP |
| 12_121416816 | C | T | rs568123980 | HNF1A | NM_000545.6 | c.245C>T | p.Thr82Met | 0.0002 | LP |
| 12_121432176 | C | T | rs754306821 | HNF1A | NM_000545.6 | c.923C>T | p.Pro308Leu | 0.0001 | LP |
| 12_121432185 | C | A | rs757574765 | HNF1A | NM_000545.6 | c.932C>A | p.Ala311Asp | 0.0002 | LP |
| 12_121437361 | G | A | rs751368921 | HNF1A | NM_000545.6 | c.1699G>A | p.Val567Ile | 0.0001 | LP |
| 12_121438953 | C | G | rs193922591 | HNF1A | NM_000545.6 | c.1854C>G | p.Ile618Met | 0.0001 | LP |
| 7_150644456 | C | T | rs199473544 | KCNH2 | NM_000238.3 | c.3112G>A | p.Val1038Met | 0.0007 | LP |
| 7_150644471 | G | A | rs199473021 | KCNH2 | NM_000238.3 | c.3097C>T | p.Arg1033Trp | 0.0002 | LP |
| 7_150644816 | C | T | rs199473011 | KCNH2 | NM_000238.3 | c.2843G>A | p.Arg948His | 0.0001 | LP |
| 7_150644888 | C | T | rs199473009 | KCNH2 | NM_000238.3 | c.2771G>A | p.Gly924Glu | 0.0001 | LP |
| 7_150645571 | G | A | rs143512106 | KCNH2 | NM_000238.3 | c.2653C>T | p.Arg885Cys | 0.0001 | LP |
| 7_150649718 | G | A | rs199472902 | KCNH2 | NM_000238.3 | c.1352C>T | p.Pro451Leu | 0.0001 | LP |
| 7_150649763 | G | A | rs199472901 | KCNH2 | NM_000238.3 | c.1307C>T | p.Thr436Met | 0.0001 | LP |
| 7_150655198 | C | T | rs199472880 | KCNH2 | NM_000238.3 | c.865G>A | p.Glu289Lys | 0.0001 | LP |
| 11_2466525 | C | T | rs199473446 | KCNQ1 | NM_000218.2 | c.197C>T | p.Ser66Phe | 0.0001 | LP |
| 11_2549168 | G | A | rs199473449 | KCNQ1 | NM_000218.2 | c.397G>A | p.Val133Ile | 0.0001 | LP |
| 11_2549253 | G | A | rs397508111 | KCNQ1 | NM_000218.2 | c.477+5G>A |  | 0.0001 | P |
| 11_2797286 | T | C | . | KCNQ1 | NM_000218.2 | c.1685+2T>C |  | 0.0001 | P |
| 11_2869033 | G | A | rs147445322 | KCNQ1 | NM_000218.2 | c.1831G>A | p.Asp611Asn | 0.0004 | LP |
| 19_11210970 | G | A | rs778284147 | LDLR | NM_000527.5 | c.139G>A | p.Asp47Asn | 0.0002 | LP |
| 19_11213441 | G | A | rs750474121 | LDLR | NM_000527.5 | c.292G>A | p.Gly98Ser | 0.0006 | LP |
| 19_11215925 | C | T | rs774723292 | LDLR | NM_000527.5 | c.343C>T | p.Arg115Cys | 0.0001 | LP |
| 19_11215926 | G | A | rs201102461 | LDLR | NM_000527.5 | c.344G>A | p.Arg115His | 0.0017 | LP |
| 19_11216181 | T | G | rs879254586 | LDLR | NM_000527.5 | c.599T>G | p.Phe200Cys | 0.0001 | LP |
| 19_11217255 | C | T | rs879254657 | LDLR | NM_000527.5 | c.709C>T | p.Arg237Cys | 0.0001 | LP |
| 19_11217310 | G | A | rs879254669 | LDLR | NM_000527.5 | c.764G>A | p.Cys255Tyr | 0.0001 | LP |
| 19_11217315 | C | T | rs200990725 | LDLR | NM_000527.5 | c.769C>T | p.Arg257Trp | 0.0015 | LP |
| 19_11221390 | G | A | rs544453230 | LDLR | NM_000527.5 | c.1003G>A | p.Gly335Ser | 0.0002 | LP |
| 19_11223983 | C | T | rs121908043 | LDLR | NM_000527.5 | c.1216C>T | p.Arg406Trp | 0.0001 | P |
| 19_11223983 | C | A | rs121908043 | LDLR | NM_000527.5 | c.1216C>A | p.Arg406Arg | 0.0001 | P |
| 19_11224036 | C | G | . | LDLR | NM_000527.5 | c.1269C>G | p.Ile423Met | 0.0001 | LP |
| 19_11224236 | G | A | rs750363970 | LDLR | NM_000527.5 | c.1384G>A | p.Val462Ile | 0.0002 | LP |
| 19_11227576 | C | T | rs730882109 | LDLR | NM_000527.5 | c.1747C>T | p.His583Tyr | 0.0006 | LP |
| 19_11227594 | G | A | rs201971888 | LDLR | NM_000527.5 | c.1765G>A | p.Asp589Asn | 0.0015 | LP |
| 19_11227612 | C | T | rs373371572 | LDLR | NM_000527.5 | c.1783C>T | p.Arg595Trp | 0.0001 | LP |
| 19_11227613 | G | A | rs201102492 | LDLR | NM_000527.5 | c.1784G>A | p.Arg595Gln | 0.0001 | LP |
| 19_11227622 | T | C | rs879255024 | LDLR | NM_000527.5 | c.1793T>C | p.Ile598Thr | 0.0001 | LP |
| 19_11230801 | G | A | rs879255066 | LDLR | NM_000527.5 | c.1879G>A | p.Ala627Thr | 0.0003 | P |
| 19_11231084 | G | C | rs745753810 | LDLR | NM_000527.5 | c.2026G>C | p.Gly676Arg | 0.0008 | P |
| 19_11231108 | G | A | rs774730452 | LDLR | NM_000527.5 | c.2050G>A | p.Ala684Thr | 0.0001 | LP |
| 1_156104594 | A | G | rs1572359505 | LMNA | NM_170707.4 | c.640-2A>G |  | 0.0001 | LP |
| 1_156106043 | G | A | rs267607563 | LMNA | NM_170707.4 | c.1196G>A | p.Arg399His | 0.0003 | LP |
| 1_156106776 | G | A | rs11575937 | LMNA | NM_170707.4 | c.1445G>A | p.Arg482Gln | 0.0001 | P |
| 1_156106994 | C | T | rs57318642 | LMNA | NM_170707.4 | c.1579C>T | p.Arg527Cys | 0.0003 | LP |
| 1_156106995 | G | A | rs57520892 | LMNA | NM_170707.4 | c.1580G>A | p.Arg527His | 0.0001 | P |
| 1_156108325 | G | A | rs57830985 | LMNA | NM_170707.4 | c.1655G>A | p.Arg552His | 0.0001 | P |
| 11_64575121 | C | A | . | MEN1 | NM_000244.3 | c.701G>T | p.Arg234Leu | 0.0001 | LP |
| 3_37067242 | C | T | rs63750760 | MLH1 | NM_000249.4 | c.1153C>T | p.Arg385Cys | 0.0006 | LP |
| 3_37067254 | C | T | rs61751644 | MLH1 | NM_000249.4 | c.1165C>T | p.Arg389Trp | 0.0001 | LP |
| 2_47630344 | C | A | rs56170584 | MSH2 | NM_000251.2 | c.14C>A | p.Pro5Gln | 0.0025 | LP |
| 2_47707892 | A | G | rs63750027 | MSH2 | NM_000251.2 | c.2516A>G | p.His839Arg | 0.0012 | LP |
| 2_47709916 | A | G | rs1114167818 | MSH2 | NM_000251.2 | c.2635-2A>G |  | 0.0001 | LP |
| 2_48027824 | G | A | rs63749889 | MSH6 | NM_000179.3 | c.2702G>A | p.Arg901His | 0.0002 | LP |
| 2_48028143 | G | A | rs587779253 | MSH6 | NM_000179.3 | c.3021G>A | p.Trp1007* | 0.0001 | P |
| 2_48030645 | C | A | rs63750998 | MSH6 | NM_000179.3 | c.3259C>A | p.Pro1087Thr | 0.0003 | LP |
| 2_48030685 | C | T | rs63750442 | MSH6 | NM_000179.3 | c.3299C>T | p.Thr1100Met | 0.0002 | LP |
| 2_48033640 | C | T | rs63750836 | MSH6 | NM_000179.3 | c.3851C>T | p.Thr1284Met | 0.0001 | LP |
| 1_45796895 | C | A | rs376790729 | MUTYH | NM_001048171.1 | c.1393G>T | p.Glu465* | 0.0001 | P |
| 1_45797139 | G | A | rs150792276 | MUTYH | NM_001048171.1 | c.1234C>T | p.Arg412Cys | 0.0001 | LP |
| 1_45797228 | C | T | rs36053993 | MUTYH | NM_001048171.1 | c.1145G>A | p.Gly382Asp | 0.0002 | P |
| 1_45797760 | T | C | rs77542170 | MUTYH | NM_001048171.1 | c.850-2A>G |  | 0.0131 | LP |
| 1_45797914 | C | T | rs730881833 | MUTYH | NM_001048171.1 | c.815G>A | p.Gly272Glu | 0.0002 | P |
| 1_45797951 | G | A | rs769237459 | MUTYH | NM_001048171.1 | c.778C>T | p.Arg260Trp | 0.0001 | P |
| 1_45797972 | G | A | rs786203115 | MUTYH | NM_001048171.1 | c.757C>T | p.Gln253* | 0.0003 | P |
| 1_45798117 | C | T | rs140342925 | MUTYH | NM_001048171.1 | c.692G>A | p.Arg231His | 0.0001 | P |
| 1_45798130 | G | A | rs34126013 | MUTYH | NM_001048171.1 | c.679C>T | p.Arg227Trp | 0.0002 | P |
| 1_45798460 | C | T | rs758567247 | MUTYH | NM_001048171.1 | c.509G>A | p.Arg170Gln | 0.0002 | LP |
| 1_45798627 | C | T | rs762307622 | MUTYH | NM_001048171.1 | c.425G>A | p.Trp142* | 0.0007 | P |
| 1_45799108 | G | A | rs765123255 | MUTYH | NM_001048171.1 | c.283C>T | p.Arg95Trp | 0.0002 | P |
| 1_45800165 | G | A | rs587780088 | MUTYH | NM_001048171.1 | c.55C>T | p.Arg19* | 0.0002 | P |
| 11_47353695 | C | T | rs202147520 | MYBPC3 | NM_000256.3 | c.3742G>A | p.Gly1248Arg | 0.0001 | LP |
| 11_47355161 | G | A | rs371061770 | MYBPC3 | NM_000256.3 | c.3137C>T | p.Thr1046Met | 0.0002 | LP |
| 11_47355304 | C | T | rs730880584 | MYBPC3 | NM_000256.3 | c.2995-1G>A |  | 0.0001 | P |
| 11_47359040 | C | A | rs527305885 | MYBPC3 | NM_000256.3 | c.2504G>T | p.Arg835Leu | 0.0013 | LP |
| 11_47370065 | C | T | rs369300885 | MYBPC3 | NM_000256.3 | c.682G>A | p.Asp228Asn | 0.0001 | LP |
| 11_47371422 | G | A | rs727503216 | MYBPC3 | NM_000256.3 | c.557C>T | p.Pro186Leu | 0.0001 | LP |
| 14_23883310 | G | A | rs372381770 | MYH7 | NM_000257.4 | c.5561C>T | p.Thr1854Met | 0.0001 | LP |
| 14_23884227 | G | A | rs12590294 | MYH7 | NM_000257.4 | c.5536C>T | p.Arg1846Cys | 0.0002 | LP |
| 14_23884594 | G | A | rs727505294 | MYH7 | NM_000257.4 | c.5279C>T | p.Thr1760Met | 0.0001 | LP |
| 14_23884860 | C | T | rs193922390 | MYH7 | NM_000257.4 | c.5135G>A | p.Arg1712Gln | 0.0001 | LP |
| 14_23884924 | C | T | rs45464193 | MYH7 | NM_000257.4 | c.5071G>A | p.Val1691Met | 0.0001 | LP |
| 14_23887607 | G | T | rs141764279 | MYH7 | NM_000257.4 | c.3981C>A | p.Asn1327Lys | 0.0001 | LP |
| 14_23891500 | C | T | rs397516178 | MYH7 | NM_000257.4 | c.3134G>A | p.Arg1045His | 0.0002 | LP |
| 14_23894051 | C | T | rs202141173 | MYH7 | NM_000257.4 | c.2606G>A | p.Arg869His | 0.0001 | LP |
| 14_23894554 | C | T | rs376754645 | MYH7 | NM_000257.4 | c.2360G>A | p.Arg787His | 0.0001 | LP |
| 14_23896042 | C | T | rs371898076 | MYH7 | NM_000257.4 | c.1988G>A | p.Arg663His | 0.0001 | P |
| 14_23898247 | G | A | rs148808089 | MYH7 | NM_000257.4 | c.1324C>T | p.Arg442Cys | 0.0001 | P |
| 14_23898249 | G | A | rs121913653 | MYH7 | NM_000257.4 | c.1322C>T | p.Thr441Met | 0.0011 | LP |
| 14_23901923 | G | A | rs727503278 | MYH7 | NM_000257.4 | c.427C>T | p.Arg143Trp | 0.0001 | LP |
| 3_46900985 | C | T | rs104893749 | MYL3 | NM_000258.3 | c.461G>A | p.Arg154His | 0.0003 | LP |
| 3_46902303 | G | C | rs139794067 | MYL3 | NM_000258.3 | c.170C>G | p.Ala57Gly | 0.0001 | LP |
| 16_23634318 | C | A | rs876659036 | PALB2 | NM_024675.3 | c.2968G>T | p.Glu990* | 0.0002 | P |
| 16_23635403 | G | GT | rs876660147 | PALB2 | NM_024675.3 | c.2760dupA | p.Gln921Thrfs*7 | 0.0002 | P |
| 16_23637612 | C | T | rs876658983 | PALB2 | NM_024675.3 | c.2693G>A | p.Trp898* | 0.0001 | P |
| 16_23647116 | G | A | rs180177091 | PALB2 | NM_024675.3 | c.751C>T | p.Gln251* | 0.0002 | P |
| 1_55505520 | G | A | rs186669805 | PCSK9 | NM_174936.3 | c.10G>A | p.Val4Ile | 0.0004 | LP |
| 1_55518082 | C | G | rs778617372 | PCSK9 | NM_174936.3 | c.655C>G | p.Gln219Glu | 0.0008 | LP |
| 1_55518371 | G | A | rs149489325 | PCSK9 | NM_174936.3 | c.706G>A | p.Gly236Ser | 0.0002 | LP |
| 1_55524243 | C | T | rs761767572 | PCSK9 | NM_174936.3 | c.1426C>T | p.Arg476Cys | 0.0001 | P |
| 1_55525195 | G | A | . | PCSK9 | NM_174936.3 | c.1540G>A | p.Ala514Thr | 0.0002 | LP |
| 1_55525219 | G | A | rs777300852 | PCSK9 | NM_174936.3 | c.1564G>A | p.Ala522Thr | 0.0002 | LP |
| 1_55527213 | C | T | rs755750316 | PCSK9 | NM_174936.3 | c.1847C>T | p.Pro616Leu | 0.0002 | LP |
| 1_55529048 | G | A | rs760437822 | PCSK9 | NM_174936.3 | c.1870G>A | p.Val624Met | 0.0001 | LP |
| 1_55529182 | C | A | rs762298323 | PCSK9 | NM_174936.3 | c.2004C>A | p.Ser668Arg | 0.0002 | LP |
| 12_32949154 | G | T | rs727504950 | PKP2 | NM_004572.3 | c.2378C>A | p.Ala793Asp | 0.0001 | LP |
| 12_32974457 | G | A | rs762753884 | PKP2 | NM_004572.3 | c.1978C>T | p.Gln660* | 0.0001 | P |
| 12_33031341 | C | T | rs397517027 | PKP2 | NM_004572.3 | c.473G>A | p.Arg158Lys | 0.0008 | LP |
| 12_33049514 | ACTGT | A | rs397516997 | PKP2 | NM_004572.3 | c.148_151delACAG | p.Thr50Serfs*61 | 0.0001 | P |
| 7_6018305 | CAGTTA | C | rs63750695 | PMS2 | NM_000535.7 | c.2192_2196delTAACT | p.Leu731Cysfs*3 | 0.0001 | P |
| 7_6026658 | T | A | rs267608169 | PMS2 | NM_000535.7 | c.1738A>T | p.Lys580* | 0.0002 | P |
| 10_89711909 | A | G | rs757498880 | PTEN | NM_000314.7 | c.527A>G | p.Tyr176Cys | 0.0001 | LP |
| 10_43601917 | G | A | rs377767388 | RET | NM_020975.6 | c.961G>A | p.Gly321Arg | 0.0001 | LP |
| 10_43606686 | C | A | rs552057730 | RET | NM_020975.6 | c.1295C>A | p.Ala432Glu | 0.0001 | LP |
| 10_43606814 | C | T | rs746512075 | RET | NM_020975.6 | c.1423C>T | p.Arg475Trp | 0.0001 | LP |
| 10_43606829 | G | A | rs537874538 | RET | NM_020975.6 | c.1438G>A | p.Glu480Lys | 0.0001 | LP |
| 10_43607621 | G | A | rs75873440 | RET | NM_020975.6 | c.1597G>A | p.Gly533Ser | 0.0002 | LP |
| 10_43609942 | G | A | rs377767407 | RET | NM_020975.6 | c.1894G>A | p.Glu632Lys | 0.0002 | LP |
| 1_68895518 | G | A | rs121917745 | RPE65 | NM_000329.3 | c.1543C>T | p.Arg515Trp | 0.0001 | P |
| 1_68910558 | C | T | rs61752870 | RPE65 | NM_000329.3 | c.254G>A | p.Arg85His | 0.0001 | LP |
| 19_38931470 | G | A | rs139161723 | RYR1 | NM_000540.2 | c.131G>A | p.Arg44His | 0.0002 | LP |
| 19_38931491 | C | A | rs193922749 | RYR1 | NM_000540.2 | c.152C>A | p.Thr51Asn | 0.0001 | LP |
| 19_38945968 | G | A | rs118192119 | RYR1 | NM_000540.2 | c.1534G>A | p.Glu512Lys | 0.0001 | P |
| 19_38948179 | G | A | rs118204423 | RYR1 | NM_000540.2 | c.1834G>A | p.Ala612Thr | 0.0007 | LP |
| 19_38958433 | A | G | rs756138074 | RYR1 | NM_000540.2 | c.3362A>G | p.Tyr1121Cys | 0.0001 | LP |
| 19_38980770 | T | A | rs192932788 | RYR1 | NM_000540.2 | c.5869T>A | p.Ser1957Thr | 0.0006 | LP |
| 19_38985105 | G | A | rs193922789 | RYR1 | NM_000540.2 | c.6388G>A | p.Gly2130Arg | 0.0001 | LP |
| 19_38985195 | G | A | rs143398211 | RYR1 | NM_000540.2 | c.6478G>A | p.Gly2160Ser | 0.0001 | LP |
| 19_38991282 | C | T | rs193922816 | RYR1 | NM_000540.2 | c.7360C>T | p.Arg2454Cys | 0.0001 | LP |
| 19_39003007 | G | A | rs193922833 | RYR1 | NM_000540.2 | c.9356G>A | p.Arg3119His | 0.0004 | LP |
| 19_39025415 | G | A | rs193922839 | RYR1 | NM_000540.2 | c.11315G>A | p.Arg3772Gln | 0.0001 | LP |
| 19_39026638 | G | A | rs140616359 | RYR1 | NM_000540.2 | c.11518G>A | p.Val3840Ile | 0.0010 | LP |
| 19_39039020 | C | T | rs373406011 | RYR1 | NM_000540.2 | c.12242C>T | p.Thr4081Met | 0.0004 | LP |
| 19_39063948 | G | A | rs142929172 | RYR1 | NM_000540.2 | c.14129+1G>A |  | 0.0002 | P |
| 19_39076780 | C | T | rs146876145 | RYR1 | NM_000540.2 | c.14918C>T | p.Pro4973Leu | 0.0003 | P |
| 1_237494239 | C | T | rs1060500142 | RYR2 | NM_001035.3 | c.230C>T | p.Ala77Val | 0.0001 | P |
| 1_237729972 | C | T | rs200236750 | RYR2 | NM_001035.3 | c.3320C>T | p.Thr1107Met | 0.0006 | LP |
| 1_237777937 | G | A | rs749184443 | RYR2 | NM_001035.3 | c.5509G>A | p.Glu1837Lys | 0.0001 | LP |
| 1_237802462 | G | A | rs727504976 | RYR2 | NM_001035.3 | c.7076G>A | p.Arg2359Gln | 0.0001 | LP |
| 1_237947538 | G | A | rs794728828 | RYR2 | NM_001035.3 | c.12526G>A | p.Val4176Met | 0.0001 | LP |
| 3_38592060 | C | T | rs199473637 | SCN5A | NM_198056.2 | c.5803G>A | p.Gly1935Ser | 0.0001 | LP |
| 3_38592125 | C | T | rs199473327 | SCN5A | NM_198056.2 | c.5738G>A | p.Arg1913His | 0.0001 | LP |
| 3_38592174 | G | A | rs45465995 | SCN5A | NM_198056.2 | c.5689C>T | p.Arg1897Trp | 0.0003 | LP |
| 3_38592237 | C | CCAT | rs1474459822 | SCN5A | NM_198056.2 | c.5623_5625dupATG | p.Met1875dup | 0.0001 | LP |
| 3_38592729 | C | T | rs199473298 | SCN5A | NM_198056.2 | c.5134G>A | p.Gly1712Ser | 0.0001 | LP |
| 3_38601865 | C | T | rs199473605 | SCN5A | NM_198056.2 | c.4018G>A | p.Val1340Ile | 0.0001 | LP |
| 3_38608013 | C | T | rs199473599 | SCN5A | NM_198056.2 | c.3727G>A | p.Asp1243Asn | 0.0001 | LP |
| 3_38616870 | C | T | rs199473596 | SCN5A | NM_198056.2 | c.3584G>A | p.Arg1195His | 0.0001 | LP |
| 3_38616898 | C | T | rs199473595 | SCN5A | NM_198056.2 | c.3556G>A | p.Ala1186Thr | 0.0009 | LP |
| 3_38616915 | G | A | rs41310765 | SCN5A | NM_198056.2 | c.3539C>T | p.Ala1180Val | 0.0033 | LP |
| 3_38620875 | C | T | rs199473195 | SCN5A | NM_198056.2 | c.3340G>A | p.Asp1114Asn | 0.0001 | LP |
| 3_38620877 | G | A | rs199473194 | SCN5A | NM_198056.2 | c.3338C>T | p.Ala1113Val | 0.0001 | LP |
| 3_38622727 | G | A | rs41311135 | SCN5A | NM_198056.2 | c.2923C>T | p.Arg975Trp | 0.0002 | LP |
| 3_38622757 | G | A | rs199473180 | SCN5A | NM_198056.2 | c.2893C>T | p.Arg965Cys | 0.0002 | LP |
| 3_38627528 | C | T | rs199473584 | SCN5A | NM_198056.2 | c.2441G>A | p.Arg814Gln | 0.0002 | LP |
| 3_38629013 | C | T | rs199473157 | SCN5A | NM_198056.2 | c.2314G>A | p.Asp772Asn | 0.0003 | LP |
| 3_38639332 | G | A | rs199473149 | SCN5A | NM_198056.2 | c.2150C>T | p.Pro717Leu | 0.0001 | LP |
| 3_38639416 | C | T | rs199473145 | SCN5A | NM_198056.2 | c.2066G>A | p.Arg689His | 0.0002 | LP |
| 3_38640472 | C | T | rs199473138 | SCN5A | NM_198056.2 | c.1960G>A | p.Glu654Lys | 0.0001 | LP |
| 3_38645490 | G | A | rs1417036453 | SCN5A | NM_198056.2 | c.1603C>T | p.Arg535* | 0.0001 | P |
| 3_38645498 | A | C | rs199473573 | SCN5A | NM_198056.2 | c.1595T>G | p.Phe532Cys | 0.0001 | LP |
| 3_38647465 | C | T | rs199473570 | SCN5A | NM_198056.2 | c.1315G>A | p.Glu439Lys | 0.0001 | LP |
| 3_38647498 | C | T | rs199473111 | SCN5A | NM_198056.2 | c.1282G>A | p.Glu428Lys | 0.0003 | LP |
| 3_38648173 | C | T | rs199473101 | SCN5A | NM_198056.2 | c.1127G>A | p.Arg376His | 0.0001 | P |
| 3_38648282 | G | A | rs199473094 | SCN5A | NM_198056.2 | c.1018C>T | p.Arg340Trp | 0.0001 | LP |
| 3_38651285 | C | T | rs199473085 | SCN5A | NM_198056.2 | c.874G>A | p.Gly292Ser | 0.0009 | LP |
| 3_38655263 | C | T | rs199473071 | SCN5A | NM_198056.2 | c.674G>A | p.Arg225Gln | 0.0003 | LP |
| 3_38663892 | C | T | rs199473062 | SCN5A | NM_198056.2 | c.481G>A | p.Glu161Lys | 0.0001 | LP |
| 3_38663937 | C | T | rs199473061 | SCN5A | NM_198056.2 | c.436G>A | p.Val146Met | 0.0001 | LP |
| 3_38671911 | C | T | rs199473054 | SCN5A | NM_198056.2 | c.283G>A | p.Val95Ile | 0.0002 | LP |
| 3_38674671 | C | T | rs199473047 | SCN5A | NM_198056.2 | c.128G>A | p.Arg43Gln | 0.0003 | LP |
| 9_101911508 | A | G | rs141259922 | TGFBR1 | NM_004612.4 | c.1433A>G | p.Asn478Ser | 0.0002 | LP |
| 3_30686237 | A | G | rs779131465 | TGFBR2 | NM_003242.6 | c.95-2A>G |  | 0.0001 | LP |
| 3_30713244 | G | A | rs780542125 | TGFBR2 | NM_003242.6 | c.569G>A | p.Arg190His | 0.0003 | LP |
| 3_30713415 | A | T | rs761231369 | TGFBR2 | NM_003242.6 | c.740A>T | p.Asp247Val | 0.0002 | LP |
| 3_30729968 | C | T | rs863223852 | TGFBR2 | NM_003242.6 | c.1489C>T | p.Arg497* | 0.0001 | P |
| 19_55663278 | C | T | rs397516357 | TNNI3 | NM_000363.5 | c.557G>A | p.Arg186Gln | 0.0001 | P |
| 19_55665463 | G | A | rs368861241 | TNNI3 | NM_000363.5 | c.484C>T | p.Arg162Trp | 0.0001 | P |
| 1_201328348 | C | T | rs141121678 | TNNT2 | NM_001276345.2 | c.887G>A | p.Arg296His | 0.0001 | LP |
| 1_201328373 | G | A | rs121964857 | TNNT2 | NM_001276345.2 | c.862C>T | p.Arg288Cys | 0.0001 | LP |
| 1_201328787 | T | C | rs397516483 | TNNT2 | NM_001276345.2 | c.815A>G | p.Asn272Ser | 0.0001 | LP |
| 1_201333455 | G | A | rs483352832 | TNNT2 | NM_001276345.2 | c.460C>T | p.Arg154Trp | 0.0001 | LP |
| 1_201333463 | C | T | rs730881101 | TNNT2 | NM_001276345.2 | c.452G>A | p.Arg151Gln | 0.0001 | LP |
| 1_201333497 | G | A | rs397516463 | TNNT2 | NM_001276345.2 | c.418C>T | p.Arg140Cys | 0.0001 | P |
| 1_201334389 | G | A | rs727504245 | TNNT2 | NM_001276345.2 | c.341C>T | p.Ala114Val | 0.0001 | LP |
| 17_7577539 | G | C | rs121912651 | TP53 | NM_000546.6 | c.742C>G | p.Arg248Gly | 0.0001 | LP |
| 17_7578406 | C | T | rs28934578 | TP53 | NM_000546.6 | c.524G>A | p.Arg175His | 0.0001 | P |
| 11_32413587 | T | C | rs751932589 | WT1 | NM_024426.6 | c.1378A>G | p.Thr460Ala | 0.0001 | LP |

**Table S3**. Statistics of the pathogenic/likely pathogenic variants in the recommendation genes

| **Phenotypes** | **Gene** | **Inheritance** | **Total variants** | **P/LP**  **variants** | **Ratio**  **of P/LP** | **Population Frequency** |
| --- | --- | --- | --- | --- | --- | --- |
| **Genes related to cancer phenotypes** | | | | | | |
| Familial adenomatous polyposis | *APC* | AD | 192 | 2 | 0.0104 | 0.0042 |
| Familial medullary thyroid cancer | *RET* | AD | 114 | 6 | 0.0526 | 0.0018 |
| Hereditary breast and/or  ovarian cancer | *BRCA1* | AD | 116 | 3 | 0.0259 | 0.0011 |
|  | *BRCA2* | AD | 232 | 14 | 0.0603 | 0.0054 |
|  | *PALB2* | AD | 84 | 4 | 0.0476 | 0.0016 |
| Hereditary paraganglioma-  pheochromocytoma syndrome | *MAX* | AD | 17 | 0 | 0.0000 | 0.0000 |
|  | *SDHB* | AD | 14 | 0 | 0.0000 | 0.0000 |
|  | *SDHD* | AD | 9 | 0 | 0.0000 | 0.0000 |
|  | *SDHAF2* | AD | 11 | 0 | 0.0000 | 0.0000 |
|  | *SDHC* | AD | 10 | 0 | 0.0000 | 0.0000 |
|  | *TMEM127* | AD | 13 | 0 | 0.0000 | 0.0000 |
| Juvenile polyposis syndrome | *BMPR1A* | AD | 21 | 0 | 0.0000 | 0.0000 |
|  | *SMAD4* | AD | 25 | 0 | 0.0000 | 0.0000 |
| Li-Fraumeni syndrome | *TP53* | AD | 34 | 2 | 0.0588 | 0.0004 |
| Lynch syndrome | *MLH1* | AD | 53 | 2 | 0.0377 | 0.0013 |
|  | *MSH2* | AD | 92 | 3 | 0.0326 | 0.0076 |
|  | *MSH6* | AD | 161 | 5 | 0.0311 | 0.0020 |
|  | *PMS2* | AD | 61 | 2 | 0.0328 | 0.0007 |
| Multiple endocrine neoplasia type 1 | *MEN1* | AD | 36 | 1 | 0.0278 | 0.0002 |
| *MUTYH*-associated polyposis | *MUTYH* | AR | 54 | 13 | 0.2407 | 0.0313 |
| Neurofibromatosis type 2 | *NF2* | AD | 26 | 0 | 0.0000 | 0.0000 |
| Peutz-Jeghers syndrome | *STK11* | AD | 41 | 0 | 0.0000 | 0.0000 |
| *PTEN* hamartoma tumor syndrome | *PTEN* | AD | 37 | 1 | 0.0270 | 0.0002 |
| Retinoblastoma | *RB1* | AD | 35 | 0 | 0.0000 | 0.0000 |
| Tuberous sclerosis complex | *TSC1* | AD | 97 | 0 | 0.0000 | 0.0000 |
|  | *TSC2* | AD | 239 | 0 | 0.0000 | 0.0000 |
| von Hippel-Lindau syndrome | *VHL* | AD | 29 | 0 | 0.0000 | 0.0000 |
| *WT1*-related Wilms tumor | *WT1* | AD | 42 | 1 | 0.0238 | 0.0002 |
| **Genes related to cardiovascular phenotypes** | | | | | | |
| Aortopathies | *ACTA2* | AD | 35 | 2 | 0.0571 | 0.0004 |
|  | *FBN1* | AD | 149 | 9 | 0.0604 | 0.0036 |
|  | *MYH11* | AD | 179 | 0 | 0.0000 | 0.0000 |
|  | *SMAD3* | AD | 27 | 0 | 0.0000 | 0.0000 |
|  | *TGFBR1* | AD | 13 | 1 | 0.0769 | 0.0004 |
|  | *TGFBR2* | AD | 41 | 4 | 0.0976 | 0.0016 |
| Arrhythmogenic right ventricular  cardiomyopathy | *DSC2* | AD | 62 | 0 | 0.0000 | 0.0000 |
|  | *DSG2* | AD | 93 | 2 | 0.0215 | 0.0033 |
|  | *DSP* | AD | 256 | 1 | 0.0039 | 0.0002 |
|  | *PKP2* | AD | 95 | 4 | 0.0421 | 0.0022 |
|  | *TMEM43* | AD | 47 | 0 | 0.0000 | 0.0000 |
| Catecholaminergic polymorphic  ventricular tachycardia | *CASQ2* | AR | 37 | 1 | 0.0270 | 0.0002 |
|  | *RYR2* | AD | 306 | 5 | 0.0163 | 0.0020 |
|  | *TRDN* | AR | 64 | 0 | 0.0000 | 0.0000 |
| Dilated cardiomyopathy | *FLNC* | AD | 267 | 0 | 0.0000 | 0.0000 |
|  | *LMNA* | AD | 72 | 6 | 0.0833 | 0.0022 |
|  | *TNNT2* | AD | 29 | 7 | 0.2414 | 0.0016 |
|  | *TTN* | AD | 2856 | 0 | 0.0000 | 0.0000 |
| Ehlers-Danlos syndrome,  vascular type | *COL3A1* | AD | 155 | 0 | 0.0000 | 0.0000 |
| Familial hypercholesterolemia | *APOB* | AD | 355 | 6 | 0.0169 | 0.0036 |
|  | *LDLR* | AD | 97 | 21 | 0.2165 | 0.0172 |
|  | *PCSK9* | AD | 85 | 9 | 0.1059 | 0.0051 |
| Hypertrophic cardiomyopathy | *ACTC1* | AD | 15 | 0 | 0.0000 | 0.0000 |
|  | *MYBPC3* | AD | 138 | 6 | 0.0435 | 0.0040 |
|  | *MYH7* | AD | 139 | 13 | 0.0935 | 0.0054 |
|  | *MYL2* | AD | 13 | 0 | 0.0000 | 0.0000 |
|  | *MYL3* | AD | 17 | 2 | 0.1176 | 0.0009 |
|  | *PRKAG2* | AD | 50 | 0 | 0.0000 | 0.0000 |
|  | *TPM1* | AD | 25 | 0 | 0.0000 | 0.0000 |
|  | *TNNI3* | AD | 24 | 2 | 0.0833 | 0.0000 |
| Long QT syndrome types 1 | *KCNQ1* | AD | 71 | 5 | 0.0704 | 0.0018 |
| Long QT syndrome types 2 | *KCNH2* | AD | 130 | 8 | 0.0615 | 0.0031 |
| Long QT syndrome 3;  Brugada syndrome | *SCN5A* | AD | 211 | 31 | 0.1469 | 0.0196 |
| **Genes related to inborn errors of metabolism phenotypes** | | | | | | |
| Biotinidase deﬁciency | *BTD* | AR | 50 | 5 | 0.1000 | 0.0025 |
| Fabry disease | *GLA* | XL | 15 | 3 | 0.2000 | 0.0000 |
| Ornithine transcarbamylase  deﬁciency | *OTC* | XL | 12 | 0 | 0.0000 | 0.0000 |
| Pompe disease | *GAA* | AR | 147 | 19 | 0.1293 | 0.0103 |
| **Genes related to miscellaneous phenotypes** | | | | | | |
| Hereditary hemochromatosis | *HFE* | AR | 43 | 0 | 0.0000 | 0.0000 |
| Hereditary hemorrhagic telangiectasia | *ACVRL1* | AD | 40 | 1 | 0.0250 | 0.0002 |
|  | *ENG* | AD | 61 | 1 | 0.0164 | 0.0002 |
| Malignant hyperthermia | *CACNA1S* | AD | 195 | 0 | 0.0000 | 0.0000 |
|  | *RYR1* | AD | 488 | 15 | 0.0307 | 0.0094 |
| Maturity-onset diabetes of  the young | *HNF1A* | AD | 71 | 6 | 0.0845 | 0.0022 |
| RPE65-related retinopathy | *RPE65* | AR | 40 | 2 | 0.0500 | 0.0004 |
| Wilson disease | *ATP7B* | AR | 163 | 39 | 0.2393 | 0.0286 |
| **Total** |  |  | **9,373** | **295** |  | **0.1737** |

AD autosomal dominant, AR autosomal recessive, XL X-linked, P pathogenic, LP likely pathogenic; Total variants: the variants in exonic, splicing sites, intron and UTR regions for each gene; P/LP Variants: the pathogenic or likely pathogenic variants in a gene; Ratio of P/LP: the proportion of P/LP variants in the total variants for each gene; Population Frequency of P/LP: the percentage of sum of individuals carrying P/LP variants for each gene in 4480 Chinese individuals.
